# Coffee Consumption and *CYP1A2* Polymorphism Involvement in Type 2 Diabetes in a Romanian Population

**DOI:** 10.3390/jpm14070717

**Published:** 2024-07-03

**Authors:** Laura Claudia Popa, Simona Sorina Farcas, Nicoleta Ioana Andreescu

**Affiliations:** 1 Department of Microscopic Morphology, Discipline of Genetics, Genomic Medicine Centre “Victor Babeș”, University of Medicine and Pharmacy, 300041 Timisoara, Romania; laura.popa@umft.ro (L.C.P.); andreescu.nicoleta@umft.ro (N.I.A.); 2 “Louis Turcanu” Children Emergency Hospital, 300011 Timisoara, Romania

**Keywords:** *CYP1A2*, rs762551, coffee intake, caffeine, type 2 diabetes, glucose, cholesterol

## Abstract

Cytochrome P450 1A2 (CYP1A2) is known to be the main enzyme directly responsible for caffeine metabolism. Rs762551 (NC_000015.10:g.74749576C>A) is a single nucleotide polymorphism of the *CYP1A2* gene, and it is known mainly for metabolizing caffeine. A significant worldwide health issue, type 2 diabetes (T2DM), has been reported to be negatively associated with coffee consumption. Yet, some studies have proven that high intakes of coffee can lead to a late onset of T2DM. Objectives: This study aims to find any significant correlations among *CYP1A2* polymorphism, coffee consumption, and T2DM. Methods: A total of 358 people were enrolled in this study—218 diagnosed with T2DM, and 140 representing the control sample. The qPCR technique was performed, analyzing rs762551 (assay C_8881221) on the LightCycler 480 (Roche, Basel, Switzerland) with Gene Scanning software version 1.5.1 (Roche). Results: Our first observation was that the diabetic patients were likely to consume more coffee than the non-diabetic subjects. People with the AA genotype, or the fast metabolizers, are the least common, yet they are the highest coffee consumers and present the highest glucose and cholesterol levels. Another important finding is the correlation between coffee intake and glucose level, which showed statistically significant differences between the diabetic group (*p* = 0.0002) and the control group (*p* = 0.029). Conclusions: The main conclusion of this study is that according to genotype, caffeine levels, glucose, and cholesterol are interconnected and proportionally related, regardless of type 2 diabetes.

## 1. Introduction

There is controversy over the relationship between coffee intake and the risk of developing type 2 diabetes. In past years, multiple publications have reported a negative association between coffee consumption and the risk of type 2 diabetes (T2DM). Yet, regular coffee consumption in exceptionally high intakes may play a role in the delayed onset of T2DM [1,2].

As one of the most popular beverages worldwide, coffee is a mixture of bioactive chemicals, each with a different effect on the modulation of glycemic levels. Two bioactive chemicals are the polyphenols dihydrocaffeic acid and chlorogenic acid, which act as antioxidants and favor glucose homeostasis [3]. Other components of coffee are caffeine, magnesium, and trigonelline [4]. It is still unclear which components have the highest protective role on glucose metabolism. The Health Professionals’ Follow-Up Study proved that caffeinated coffee intake leads to a 4% lower risk of T2DM, while decaffeinated coffee leads to a 7% lower risk of T2DM [5]. Considering the protective role of decaffeinated coffee [6], we can assume that the other components, rather than the caffeine, might also interfere with glucose metabolism.

Diabetes mellitus is a well-known major health issue worldwide that raises high interest due to its increased morbidity and mortality [1]. It is a group of metabolic diseases defined by abnormal insulin secretion or insulin resistance, resulting in persistent hyperglycemia [7,8].

“Metabolic syndrome” associates insulin resistance, obesity or a high body mass index, elevated blood pressure, and dyslipidemia. A large number of studies have proven the association between metabolic syndrome cardiovascular diseases and type 2 diabetes mellitus [9].

Type 2 diabetes mellitus (T2DM) represents 90% of all diabetes cases [8], and it is by far the most common and aggressive type of diabetes. T2DM is defined by a decreased response to insulin, also known as insulin resistance [10]. Genetic predisposition, a sedentary lifestyle, and excessive caloric intake are well-known trigger factors for T2DM [11]. Still, recent studies have shown that several genes have also been functionally involved in the pathogenesis of T2DM [12].

The cytochrome P450 gene superfamily encodes many enzymes, with more than 150 known [13]. Their primary role has been demonstrated to catalyze many chemical reactions [14]. Cytochrome P450 1A2 (CYP1A2) almost entirely metabolizes caffeine in the body [15].

CYP1A2 is mainly expressed in the liver [16]. The enzyme encodes monooxygenase, which is implicated in drug metabolism, cholesterol, steroids, and the synthesis of other lipids [7,17].

*CYP1A2* presents a wide variability among individuals, regulated by a genetic polymorphism [18]. Homozygous individuals for the *CYP1A2* *1A/*1A genotype are fast caffeine metabolizers, while homozygous carriers for the e *1F allele (or the C polymorphism) are slow caffeine metabolizers. Consequently, they are exposed to caffeine for extended periods [19]. Coffee consumption and insulin act as inducers of CYP1A2 enzyme activity [20]; it is also known that regular coffee consumption reduces the risk of prediabetes and T2DM in connection with genetic polymorphisms [21,22]. However, the relationships among *CYP1A2*, coffee consumption, and T2DM are still unclear.

From a microscopic point of view, an essential factor in T2DM pathogenesis is insulin resistance, which is influenced by diet and other living habits [23]. Even though coffee consumption shows no effect on metabolic syndrome effects [24], it has been proven that caffeine consumption can acutely boost insulin resistance [25]. However, the mechanisms that reduce glucose levels and, as a result, lower the risk of T2DM after consuming coffee, are not well understood [25,26].

Euglycemic properties are responsible for the effect of caffeine on blood glucose, which reduces short-term insulin sensitivity while suppressing muscle glycogenesis due to high epinephrine discharge [27].

To find any significant correlations among *CYP1A2* polymorphism, coffee consumption, and T2DM, we studied a T2DM group and compared the results with those from a control group. We analyzed these subjects’ *CYP1A2* polymorphisms with coffee consumption and other biochemical parameters.

A Mendelian randomization study demonstrated that almost half of the caffeine’s effect on T2DM was estimated to be mediated by lowering the BMI, which substantially impacted T2DM and other cardiovascular diseases [28].

Thus, the mechanism by which coffee consumption decreases the risk of T2DM is unclear. However, studies have demonstrated that drinking coffee may play a role in preventing inflammatory and oxidative stress-related diseases, such as obesity, metabolic syndrome, and T2DM [29].

Also, an alkaloid in coffee, trigonelline, is known for its anti-hyperglycemic effect [30].

## 2. Materials and Methods

### 2.1. Subjects

According to the National Center for Statistics and Informatics in Public Health 2017, there were 1,785,300 diabetic patients from a registered adult population of 14,382,000 people in Romania. This statistically means that 12.5% of Romania’s adult population (20–79 years) was suffering from diabetes mellitus.

Regarding the regional distribution, 8.2% of the total diabetic Romanian population was registered in the western region of Romania, including Timis County, more likely affecting women than men. According to Timis Public Health, in 2017, there were 47,067 diabetic patients under observation in Timis County [31]. Given the presented population data, we were able to calculate the sample size for this study to meet the criteria of a 95% confidence level, a 5% margin of error, and a 50% population proportion.

A total of 358 adult patients matching the metabolic syndrome criteria were initially recruited; 218 were diagnosed with type 2 diabetes mellitus, with 198 of them being under oral antidiabetic treatment at the time the study took place, and 140 subjects not presenting type 2 diabetes. The inclusion criteria were represented by obesity, with a BMI above 25, high triglycerides, low HDL cholesterol, high blood pressure, and high blood glucose after overnight fasting.

The patients with T2DM were diagnosed according to the World Health Organization’s criteria (fasting plasma glucose values of ≥7.0 mmol/L (126 mg/dL), 2 h post-load plasma glucose of ≥11.1 mmol/L (200 mg/dL) in the presence of signs and symptoms of diabetes) [32]. The 140 subjects with no T2DM or presenting other forms of diabetes represented this study’s control group.

All participants signed informed consent forms and authorization for blood sampling and banking. The present study is part of a much larger investigation, and the patients were informed and consented to participate. The investigation has conformed to the Declaration of Helsinki and institutional guidelines and has been approved by the Medical Ethical Committee of Victor Babes University.

Blood samples were taken in the morning (between 8 am and 10 am) into EDTA purple vacutainer tubes after 12–14 h of fasting. A 4 mL blood sample was collected from each patient. The biochemical testing was performed in a private accredited laboratory, while the genetic testing was performed at the Regional Genomics Center Timis.

The amount of coffee consumption per day was determined using questionnaires, with responses to the following questions: “How much coffee do you drink per day (in ml)?”, “Is the coffee that you drink caffeinated or decaffeinated?”, and “Do you add milk to your coffee? How much?”.

A 24 h diet recall, administered by trained medical physicians or volunteer medical students, determined the daily nutrient intake. A 24 h recall is a dietary evaluation tool structured as an interview in which participants are asked to recollect all the food and beverages they have consumed during the last 24 h, using the 5-pass approach. The 5-pass approach consists of the following: (1) a list of food and drinks consumed 24 h prior is composed by the subject; (2) the most frequently forgotten foods (such as sauce or drinks consumed between meals) are documented; (3) the times and occasions when the food and drinks were consumed; (4) descriptions of each food and the amounts involved; (5) a final review of the list [33].

When completing the questionnaires, the research staff paid more attention to coffee consumption, emphasizing the type and amount of coffee consumed, whether they added sugar or milk, and the amounts added. All these details play an essential role in calculating the final caffeine level.

For each participant, the diet recall was applied on four non-consecutive days, with a face-to-face interview for the first recall and a telephone interview for the second, third, and fourth interviews. The participants were interviewed in four sessions to determine the flexibility or consistency of their dietary intake.

We calculated each participant’s caffeine consumption using the Nutrio web application (Naturalpixel SRL, Bucharest, Romania, https://nutritioapp.com). For each day investigated, the amounts of food and drinks declared by the participant were converted into energy, macro-, and micro-nutrients. As expected, we observed that beverages such as coffee and tea were the primary sources of caffeine.

The correlation parameters also included age, gender, coffee amount, caffeine amount, glucose value, and body mass index (BMI).

### 2.2. SNP Selection and Genotyping

According to the manufacturer’s procedures of the MagCore Nucleic Acid Extraction Kit (RBC Bioscience, New Taipei City, Taiwan), DNA was extracted from the whole blood samples. Afterward, the DNA concentration was determined using an Epoch Microplate Spectrophotometer (Agilent BioTek EPOCHSN, Santa Clara, CA, USA).

For this study, we chose to analyze one SNP in the *CYP1A2* gene, rs762551 (assay C_8881221), because it is the most associated SNP with caffeine modulation and T2DM. Genotyping of the *CYP1A2* polymorphism rs762551 was performed using the TaqMan Real-Time PCR assay (Supplied by Life Technologies Applied Biosystems, Thermo Fisher Scientific, 168 Third Avenue, Waltham, MA, USA). The detailed procedure is available in the TaqMan1 SNP genotyping assays protocol booklet provided by Applied Biosystems (ThermoFisher Scientific, 168 Third Avenue, Waltham, MA, USA).

Purified DNA was amplified in a real-time PCR reaction in the LightCycler 480 (Roche) with Gene Scanning software version 1.5.1 (Roche) using a 96-well reaction plate. A reaction volume of 20 uL was used for all experiments to ensure a uniform DNA concentration in all the samples.

The Taq-Man genotyping assay we used contained two primers for amplifying the sequence of interest and two Taq-Man Minor Groove Binder (TaqMan MGB) probes for detecting alleles based on the change in fluorescence of the dyes associated with the probes.

When the PCR amplification was finished, a real-time PCR instrument performed an endpoint plate read. Fluorescence measurements (Rn) were made during the plate read, and Sequence Detection System (SDS) software (ThermoFisher Scientific, CA, USA) was used to determine and plot the Rn values based on the fluorescence signals from each well.

Two laboratory personnel performed the genotyping process in a double-blinded manner. Five percent of the samples were randomly chosen for quality control; the genotyping of these samples was repeated with 100% reproducibility.

### 2.3. Data Analysis

IBM SPSS Statistics for Windows software, Version 29.0, Armonk, NY: IBM Corp., was used to process the study data statistically. Nominal data were presented as the absolute frequency and percentage, and continuous variables were expressed as the mean and standard deviation.

The associations among the categorical variables were analyzed using cross-tabulation and the χ2 (chi-square) test. Fisher’s exact test was used if the chi-square test results were sufficiently altered and could not be considered.

The independent sample *t*-test was used to compare the means according to the dichotomous variables in the study. We used the ANOVA test to compare three or more group means where the participants were the same in each group. A statistical significance coefficient value of *p* < 0.05 was considered significant.

#### 2.3.1. Binomial Logistic Regression

Logistic regression identifies the variables that collectively distinguish cases belonging to different categories of a nominal (or categorical) variable. Binomial logistic regression is used if there are only two categories of variables to be predicted. The predictors (i.e., the independent variables) can be numeric, nominal (or categorical), or a combination of the two categories.

#### 2.3.2. OR (Odds Ratio)

The odds ratio represents the estimated relative risk, and it is used to see if the probability of an event is the same in 2 groups.

## 3. Results

### 3.1. Description of the Sample Size

**Figure 1:**
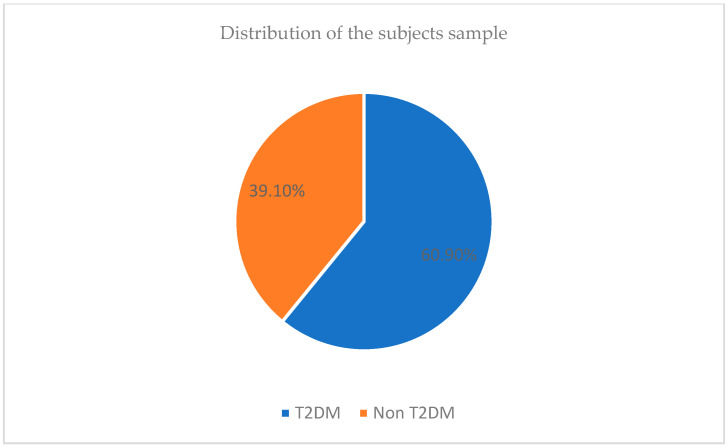
Distribution of the sample subjects.

This study was conducted on 358 patients—218 (60.9%) diagnosed with type II diabetes, and 140 (39.1%) non-diabetic subjects, as represented in the Figure 1.

The table below shows the frequencies and shares of the sample subjects according to gender, cups of coffee consumed, *CYP1A2* rs762551 genotype, and the presence of obesity. The data are reported at the level of the entire studied sample and separately for the two groups (diabetic subjects and non-diabetic).

Significantly different shares were observed between the two groups in terms of gender and the presence of obesity. The group of diabetic subjects had a higher proportion of women (54.6%) compared to the control group, where the proportion of women was only 42.9%. Also, in the group of diabetic patients, the share of people with obesity (87.2%) was significantly higher (*p* < 0.001) than in the control group (67.1%).

Table 1 also shows the mean values and standard deviations for age, coffee consumption, caffeine intake, glucose level, and cholesterol level.

The average age of the diabetic subjects (54.65 years) was significantly higher (*p* = 0.004) than that of the control group (50.92 years).

The average glucose level in patients presenting diabetes (170.74) was significantly higher than in the non-diabetic group (141.97).

Below, in Table 2 are represented the average values for age, amount of coffee consumed, caffeine, glucose, and cholesterol, and the proportion of genders divided by genotype. The three genotypes showed no significant differences in the parameters mentioned.

As shown in Table 3, we analyzed all the indicators according to genotype for the group of diabetic patients. Within this group, no significant differences were observed among the genotypes regarding the values of the analyzed parameters (gender, age, amount of coffee consumed, caffeine, glucose, cholesterol, and obesity).

As shown in the following Table 4, we tried to identify if there was any correlation between coffee intake and glucose level in diabetic patients using a one-way ANOVA test. The test proved a statistically significant difference (*p* = 0.0002) between the two variables (coffee intake and glucose level).

The same analysis was performed on non-diabetic subjects, as seen in Table 5 Yet, no significant differences among the genotypes were observed regarding the values of the analyzed parameters (gender, age, amount of coffee consumed, caffeine, glucose, cholesterol, and obesity).

Using a one-way ANOVA test, the following table (Table 6) represents the correlation between coffee intake and glucose level in non-diabetic patients. The test proved a statistically significant difference (*p* = 0.029) between the two variables (coffee intake and glucose level).

In Table 7 there are the mean values standard deviations of the following parameters:coffee consumption, caffeine, glucose, cholesterol, and the presence or absence of diabetes for combinations of two genotypes (AA + AC, AA + CC, AC + CC).

The results show similar parameters values the three analyzed groups.

#### 3.1.1. Logistic Regression

As we explained at the beginning of the statistical report, logistic regression identifies the variables that collectively distinguish the cases belonging to different categories of a nominal (or categorical) variable. Binomial logistic regression is used if there are only two categories of variables to be predicted.

#### 3.1.2. Logistic Regression Regarding the Genotype and Coffee Relation

We analyzed the prediction of coffee consumption according to the predictor variable “genotype” (belonging to the AA genotype group or belonging to the AC + CC genotype group) using multinomial logistic regression (the varying amounts of coffee, with three categories: “<1 cup”, “1–3 cups” and “>3 cups”).

The variable genotype (i.e., belonging to one of the two groups: the AA genotype or the AC + CC genotypes) did not prove to be a significant factor in predicting coffee consumption, as seen in the Table 8.

#### 3.1.3. Genotype and the Presence of Diabetes

We also analyzed the prediction of belonging to one of the two groups (diabetic or non-diabetic) according to the predictor variable genotype (i.e., either the AA, AC, or CC genotype).

The genotype, however, was not a significant predictor for diabetes, as seen in Table 9.

#### 3.1.4. Diabetes and Coffee Intake

We analyzed the diabetes prediction according to the number of cups of coffee consumed. Still, the amount of coffee was not a significant factor in predicting the occurrence of diabetes, as it can be noticed in Table 10, where *p* > 0.05.

## 4. Discussion

Type 2 diabetes is a complex, multifactorial disease with a severe impact on the world’s mortality, morbidity, and economy. In Romania, statistics show that in 2012, the prevalence of diabetes mellitus among the adult population was 9.3%, ranking Romania above the global average of 8.3% [34]. At an international level, it is estimated to affect about 629 million people worldwide by the year 2045 [35]. This study is the first to demonstrate the effects of *CYP1A2* gene polymorphisms on T2DM susceptibility in a Romanian population.

Previous studies have shown that high habitual coffee consumption is inversely proportional to the risk of T2DM prevalence [36].

A 2021 study stated that most research analyzing the association between coffee drinking and T2DM needs proof of a cause–effect relationship. They proved that habitual coffee consumption may lower the risk of type 2 diabetes by preventing the deterioration of liver and beta cell function [24]. Compared to the control group, the T2DM patients we studied consumed higher amounts of coffee, which, according to Kolb H. et al., should have protected them from the onset of the disease.

Robertson et al. [37] conducted a randomized study investigating the effects of habitual coffee intake on glucose level and lipid metabolism, involving rs762551 SNP in the *CYP1A2* gene. The interventional study intended to measure the glucose baseline and NEFA (non-esterified fatty acid) before and after coffee intake. The AC genotype patients presented a higher glucose baseline and NEFA than those with AA. After coffee intake, the AC genotype patients’ glycemic level and NEFA decreased. Our study had a retrospective design, and even though it was not interventional, we observed that coffee intake and glucose levels were directly proportionated. In addition, in our study, the AA genotype patients had the highest glucose levels, followed by the AC and CC genotype patients; the glucose levels were determined after 12 h of fasting.

Ding et al. gathered data from a meta-analysis of 28 cohort and case–control studies, and they concluded that the subjects who consumed 5 cups/day of coffee had a 30% lower risk of T2DM than the non-consumers. The associations have not presented any differences between men and women [2]. In our study, the average daily coffee amount was 140.52 mL, representing approximately 2 cups/day. When comparing the two groups of subjects, the patients suffering from T2DM proved to have consumed more coffee than the non-diabetic, yet it appears that coffee drinking did not prevent the onset of T2DM.

A cross-over randomized intervention study involving 18 volunteers examined the effects of coffee on dietary intake, appetite, and the hormones leptine and asprosin; the participants were stratified according to the −163C>A (rs762551) polymorphism of *CYP1A2* in AA (rapid metabolizers), CC (slow metabolizers), and AC (intermediate metabolizers). This study determined that rapid metabolizers (AA) were related to higher coffee consumption than slow metabolizers and associated with a lower BMI [38]. We also found that the AA genotype, or the fast metabolizers, consumed the highest amounts of coffee but presented the lowest percentage of obesity. Also related to coffee consumption, three prospective cohort studies have suggested a considerable association between moderate and high consumers of coffee and a lower risk of type 2 diabetes mellitus [39,40,41]. When studying the T2DM and control groups, diabetic patients have a higher average mean of coffee consumed, which disagrees with the anterior findings. Moreover, when dividing the whole study into genotype groups, the high coffee consumers, in our case, are the AA genotype patients, who consume the most significant amount of coffee and have the highest glucose levels.

A cross-sectional study of middle-aged Japanese men [42], including 139 cases of IFG (impaired fasting glycemia), 421 of IGT (impaired glucose tolerance), 180 of type 2 diabetes, and a control group with normal glucose tolerance, studied two different SNPs: *CYP1A2* -3860G>A (CYP1A2*1C, rs2069514) and *CYP1A2* -163C>A (CYP1A2*1F, rs762551). Neither of them showed a measurable association with IFG/IGT or type 2 diabetes. Within the -163C>A control group, the allele frequency was AA (43%), AC (44%), and CC (13%), results that are very different from what we found in the diabetic group with the following frequency order: AC, CC, and AA. Our study was slightly different from the research mentioned above. Still, as the glucose level was the common point, we can affirm that we found an association between genotype and glucose level, namely a correlation between coffee intake and glucose level in the two groups separately (diabetic and non-diabetic). The results show statistically significant differences for the diabetic group (*p* = 0.0002) and also for the the control group (*p* = 0.029).

In search of whether there is any relationship between coffee intake and the risk of T2DM, Platt et al. [43] conducted a study on 7607 subjects for which they analyzed five different *CYP1A2* SNPs, among which was our SNP rs762551. Among other findings, their study proved that rs762551 and rs2472304 showed no interaction with coffee consumption but directly increased the risk of T2DM. Our research found a correlation between coffee intake and glucose level; the results show statistically significant differences between the diabetic group (*p* = 0.0002) and the control group (*p* = 0.029).

In 2021, a study from Üsküdar University [44], based on the genotyping from the saliva of 30 healthy individuals, observed the following stratification: AA (40%), AC (50%), and CC (10%) carriers. The disadvantage of our study is that we did not perform caffeine saliva testing, and our study was based on questionnaires. Both in the control population and diabetic population, the classification was the following: AC, CC, followed by the least common genotype, AA, which is the exact opposite of what the previous study found in the general population.

Even in the absence of genotyping, Mattias Carlstrom et al. [1] conducted a meta-analysis including 30 prospective studies with 1,185,210 participants, and they concluded that for each cup-per-day increase in coffee consumption, the risk of T2D decreased by 6%. Our analysis also proves a connection between coffee intake and type 2 diabetes. Still, in the group where the coffee intake was higher, the prevalence of type 2 diabetes was also higher, but a correlation between coffee intake and glucose level was present.

Yafen Yang et al. [7] genotyped seven SNPs in *CYP19A1* and *CYP1A2*, including rs762551, for 512 T2DM patients and 515 non-diabetic controls belonging to the Chinese Han population. As a result, they found that only one SNP, namely the GC genotype of rs1062033 in CYP19A1, was significantly associated with a decreased risk of T2DM. Unfortunately, we genotyped only one SNP, the rs762551, but we did not find a statistical significance regarding the genotype (*p* = 0.583).

Paolo Palattini. et al. [3] conducted a study in a cohort of 1180 participants, in whom they investigated whether baseline coffee consumption was associated with a risk of impaired glucose tolerance. The study concluded that the heavy coffee drinkers (≥4 cups/day) who were carriers of the slow *1F allele had a higher adjusted risk of impaired fasting glucose than the non-drinking participants. In contrast, the homozygous for the *1A allele participants did not show a significant increase in risk, unrelated to if they were heavy or moderate (fewer than 4 cups/day) coffee drinkers. The study showed results very similar to ours. We also observed that heavy coffee drinkers (based on the mean average) had the highest glucose level in the control population, namely the AA genotype. Still, in the diabetic population, the AA genotype was also the highest among coffee consumers, yet the AC genotype presented the highest glucose level.

Conducting a meta-analysis to illustrate the association between habitual coffee intake and the CYP1A2 *rs762551* polymorphism, Denden et al. [45] concluded that AA genotype was firmly associated with higher coffee intake. The study was mainly oriented on gender, ethnicity, and the AA genotype and coffee intake were found only within male, younger, and Caucasian subjects, and not in female, older, and Asian subjects. Genotyping stratification results are similar to our study; we also found that the AA genotype has the highest coffee intake, but our group criteria were not so strict, and we did not separate them by gender, race and age.

### Study Strengths and Limitations

The first possible limitation of this study is the relatively small number of subjects. Still, according to the sample size calculator, the sample size is representative of the Timis County diabetic population.

A second limitation could be the average mean age of the group, which is not representative of type 2 diabetes. We look forward to extending our study in this direction to increase the value of the present finding by investigating a younger population with T2DM.

Another possible limitation could be genotyping only one SNP due to the limited financial resources. It would have been interesting to compare the results of two or three SNPs on the same population sample, but this is a future project that our team has planned.

In addition, the amount of coffee consumed by each patient was determined by questionnaires based on a 24 h diet recall. Despite the clarity of the questions and the trained personnel gathering the data, the patients may not have provided the most accurate answers. Considering this, in the future, we plan to determine the caffeine level from saliva.

## 5. Conclusions

The present study demonstrates associations among *CYP1A2* gene polymorphisms, coffee intake, glucose, and cholesterol levels among the Romanian population. We have proven that caffeine, glucose, and cholesterol are interconnected at different levels according to genotype.

In both the diabetic and non-diabetic populations we studied, the most common genotype category was the AC heterozygous genotype, or intermediate metabolizers, followed by the CC slow metabolizer genotype and the least common, the AA, or fast metabolizers.

When analyzing glucose and cholesterol levels in both diabetic and non-diabetic populations, the AA genotype had the highest levels of both parameters, followed by the CC genotype and AC genotype. In conclusion, fast metabolizers are the least common genotype, yet the highest coffee consumers and present the highest glucose and cholesterol levels.

The first conclusion of this study is that coffee intake, glucose, and cholesterol levels are proportionally related.

Another important aspect of this study is the correlation between coffee intake and glucose levels, which showed statistically significant differences between the diabetic group (*p* = 0.0002) and the control group (*p* = 0.029).

Since the statistical findings show no differences between the diabetic and the non-diabetic population, we can affirm that the *CYP1A2* polymorphisms are related to coffee intake, glucose, and cholesterol, regardless of type 2 diabetes. The only viable difference between the two groups is that the amount of coffee consumed was higher in the diabetic population.

Even though a causal effect is unclear, regular coffee consumption might have an important role in our daily lives. Unlike other studies, our investigation shows that patients who consumed larger quantities of coffee presented the highest glucose and cholesterol levels. Since this was a retrograde study and not interventional, we cannot affirm that coffee intake determines increasing glucose levels or that it is a negative predictive factor in T2DM onset.

This is the first study of habitual coffee consumption in a Romanian population related to *CYP1A2* polymorphism.

## Figures and Tables

**Table 1:** Frequencies and shares of the sample subjects according to gender, cups of coffee consumed, *CYP1A2* rs762551 genotype, and the presence of obesity, divided into three groups representing the whole sample, the diabetes subjects, and the non-diabetic subjects.

	Sample			*p*
Parameters	Whole (n = 358) Sample	Diabetes (n = 218)	Control (n = 140)	
Gender—n (%)				
Males	179 (50.0%)	99 (45.4%)	80 (57.1%)	0.030
Females	179 (50.0%)	119 (54.6%)	60 (42.9%)	
Age—mean (±SD)	53.19 (±13.15)	54.65 (±11.00)	50.92 (±13.15)	0.004
Coffee (medium amount)—mean (±SD)	129.18 (±117.04)	134.70 (±130.96)	120.58 (±91.01)	0.266
Coffee cups—n (%)				
- 1 cup	106 (29.6%)	68 (31.2%)	38 (27.1%)	0.161
- 1–3 cups	199 (55.6%)	113 (51.8%)	86 (61.4%)	
- >3 cups	53 (14.8%)	37 (17.0%)	16 (11.4%)	
Caffeine—mean (±SD)	99.52 (±115.37)	100.16 (±110.97)	98.52 (±122.30)	0.896
Genotype CYP1A2 rs762551—n (%)				
- AA	65 (18.2%)	43 (19.7%)	22 (15.7%)	0.583
- AC	163 (45.5%)	99 (45.4%)	64 (45.7%)	
- CC	130 (36.3%)	76 (34.9%)	54 (38.6%)	
Glucose—mean (±SD)	159.49 (±67.95)	170.74 (±63.50)	141.97 (±71.12)	<0.001
Obesity—n (%)				
- no	74 (20.7%)	28 (12.8%)	46 (32.9%)	<0.001
- yes	284 (79.3%)	190 (87.2%)	94 (67.1%)	
Cholesterol—mean (±SD)	313.15 (±186.11)	305.58 (±169.92)	325.12 (±209.24)	0.335

**Table 2:** Average age, coffee intake, caffeine, glucose, and cholesterol were analyzed according to each genotype.

	Genotype			*p*
Parameters	AA (n = 65)	AC (n = 163)	CC (n = 130)	
Gender—n (%)				
Male	33 (50.8%)	83 (50.9%)	63 (48.5%)	0.908
Female	32 (49.2%)	80 (49.1%)	67 (51.5%)	
Age—mean (±SD)	50.18 (±13.86)	53.61 (±11.55)	54.16 (±11.42)	0.077
Coffee (medium amount)—mean (±SD)	151.33 (±142.75)	132.91 (±117.18)	113.42 (±100.19)	0.088
Caffeine—mean (±SD)	106.06 (±119.56)	102.33 (±127.12)	92.62 (±96.60)	0.684
Glucose—mean (±SD)	167.08 (±92.76)	158.39 (±66.28)	157.07 (±54.38)	0.602
Cholesterol—mean (±SD)	348.49 (±229.90)	290.90 (±167.27)	323.74 (±182.39)	0.080

**Table 3:** Gender, age, coffee intake, caffeine, glucose, cholesterol, and obesity in diabetic patients.

Type 2 Diabetes Subjects	Genotype			*p*
Parameters	AA (n = 43)	AC (n = 99)	CC (n = 76)	
Gender—n (%)				
Male	19 (44.2%)	48 (48.5%)	32 (42.1%)	0.691
Female	24 (55.8%)	51 (51.5%)	44 (57.9%)	
Age—mean (±SD)	52.51 (±12.97)	55.52 (±10.06)	54.72 (±10.96)	0.328
Coffee (medium amount)—mean (±SD)	155.00 (±164.26)	143.24 (±127.18)	112.08 (111.92)	0.156
Caffeine—mean (±SD)	110.70 (±137.15)	99.15 (±114.51)	95.43 (±88.46)	0.768
Glucose—mean (±SD)	169.02 (±67.95)	174.10 (±69.42)	167.33 (±63.50)	0.770
Cholesterol—mean (±SD)	333.06 (±196.99)	283.13 (±161.04)	319.26 (±163.19)	0.188
Obesity—n (%)				
No	7 (16.3%)	13 (13.1%)	8 (10.5%)	0.662
Yes	36 (83.7%)	86 (89.5%)	68 (89.5%)	

**Table 4:** Correlation between coffee intake and glucose level in diabetic patients.

Groups	Sample Size	Sum	Mean	Variance		
Glucose (mg/dL)	218	37,221	170.738532	4032.44284		
Coffee amount (mL)	218	29,365.35	134.70344	17,151.6284		
Variance analysis						
Sources of variations	Sum of squares	Degree of liberty	Mean of squares	F	*p*-value	Critical value of F
Between groups	141,539.534	1	141,539.534	13.3628265	0.00028812	3.8629743
Within groups	4,596,943.45	434	10,592.0356			
Total	4,738,482.99	435				

**Table 5:** Gender, age, coffee intake, caffeine, glucose, cholesterol, and obesity in non-diabetic patients.

Control Sample	Genotype			*p*
Parameters	AA (n = 22)	AC (n = 64)	CC (n = 54)	
Gender—n (%)				
Male	14 (63.6%)	35 (54.7%)	31 (57.4%)	0.764
Female	8 (36.4%)	29 (45.3%)	23 (42.6%)	
Age—mean (±SD)	45.64 (±14.70)	50.67 (±13.07)	53.37 (±12.11)	0.065
Coffee (medium amount)—mean (±SD)	144.17 (±89.78)	116.93 (±98.59)	115.30 (±81.85)	0.417
Caffeine—mean (±SD)	97.00 (±76.23)	107.21 (±145.15)	88.66 (±107.83)	0.718
Glucose—mean (±SD)	163.27 (±130.25)	134.09 (±53.04)	142.63 (±54.18)	0.253
Cholesterol—mean (±SD)	380.08 (±288.85)	302.92 (±177.08)	330.15 (±208.35)	0.335
Obesity—n (%)				
No	8 (36.4%)	20 (31.3%)	18 (33.3%)	0.903
Yes	14 (63.6%)	44 (68.8%)	36 (66.7%)	

**Table 6:** Correlation between coffee intake and glucose level in non-diabetic patients.

Groups	Sample Size	Sum	Mean	Variance		
Glucose (mg/dL)	140	19,876	141.971429	5058.3445		
Mean amount (mL)	140	16,882.1	120.586429	8283.36179		
Variance analysis						
Sources of variations	Sum of squares	Degree of liberty	Mean of squares	F	*p*-value	Critical value of F
Between groups	32,012.2758	1	32,012.2758	4.79882783	0.02930949	3.87512601
Within groups	1,854,497.17	278	6670.85315			
Total	1,886,509.45	279				

**Table 7:** Coffee intake, caffeine, glucose, cholesterol, and the presence or absence of diabetes for combinations of two genotypes.

	Genotype		
Parameters	AA + AC (n = 228)	AA + CC (n = 195)	AC + CC (n = 293)
Coffee (medium amount)—mean (±SD)	138.16 (±124.96)	126.06 (±117.12)	124.26 (±110.21)
Caffeine—mean (±SD)	103.40 (±124.75)	97.15 (±104.77)	98.05 (±114.57)
Glucose—mean (±SD)	160.87 (±74.68)	160.41 (±69.48)	157.81 (±61.19)
Cholesterol—mean (±SD)	307.14 (±188.32)	331.94 (±199.14)	305.41 (±174.57)
Diabetes—n (%)			
No	86 (37.7%)	76 (39.0%)	118 (40.3%)
Yes	142 (62.3%)	119 (61.0%)	175 (59.7%)

**Table 8:** Correlation between coffee intake measured in cups and the genotype.

Model Fitting Information									
Model		Model Fitting Criteria				Likelihood Ratio Tests			
		−2 Log Likelihood				Chi-Square		df	Sig.
Intercept Only		23.960							
Final		19.621				4.339		2	0.114
Pseudo R-Square									
Cox and Snell						0.012			
Nagelkerke						0.014			
McFadden						0.006			
Likelihood Ratio Tests									
Effect		Model Fitting Criteria				Likelihood Ratio Tests			
		−2 Log Likelihood of Reduced Model				Chi-Square		df	Sig.
Intercept		19.621 ^a^				0.000		0	.
Genotype		23.960				4.339		2	0.114
The chi-square statistic is the difference in −2 log-likelihoods between the final and reduced models. The reduced model is formed by omitting an effect from the final model. The null hypothesis is that all parameters of that effect are 0.									
Parameter Estimates									
Coffee cups		B	Std. Error	Wald	df	Sig.	Exp(B)	95% Confidence Interval for Exp(B)	
								Lower Bound	Upper Bound
1–3 cups	Intercept	0.543	0.130	17,314	1	0.000			
	(rs = 0)	0.556	0.346	2.586	1	0.108	1.744	0.885	3.434
	(rs = 1)	0 ^b^	.	.	0	.	.	.	.
>3 cups	Intercept	−0.844	0.189	19,911	1	0.000			
	(rs = 0)	0.844	0.435	3.755	1	0.053	2.325	0.990	5.458
	(rs = 1)	0 ^b^	.	.	0	.	.	.	.
The reference category is: 1 cup equals 70 mL.									

^a^. This reduced model is equivalent to the final model because omitting the effect does not increase the degrees of freedom. ^b^. This parameter was set to zero because it is redundant.

**Table 9:** Logistic regression for the relationship between genotype and the presence/absence of diabetes.

Variables Not in the Equation							
					Score	df	Sig.
Step 0	Variables		Genotype		0.923	1	0.337
	Overall Statistics				0.923	1	0.337
Omnibus Tests of Model Coefficients							
			Chi-square			df	Sig.
Step 1	Step		0.936			1	0.333
	Block		0.936			1	0.333
	Model		0.936			1	0.333
Model Summary							
Step 1	−2 Log likelihood		Cox and Snell R Square			Nagelkerke R Square	
	478,226 ^a^		0.003			0.004	
Variables in the Equation							
		B	S.E.	Wald	df	Sig.	Exp(B)
Step 1 ^b^	Genotype	−0.276	0.288	0.919	1	0.338	0.759
	Constant	0.670	0.262	6.536	1	0.011	1.955

^a^. Estimation terminated at iteration number 3 because parameter estimates changed by less than 001. ^b^. Variable(s) entered on step 1: Genotype.

**Table 10:** Logistic regression for the relationship between the presence/absence of diabetes and coffee intake.

Variables Not in the Equation							
					Score	df	Sig.
Step 0	Variables	Coffee cups			3.654	2	0.161
		Coffee cups (1)			0.671	1	0.413
		Coffee cups (2)			3.178	1	0.075
	Overall Statistics				3.654	2	0.161
Omnibus Tests of Model Coefficients							
		Chi-square				df	Sig.
Step 1	Step	3704				2	0.157
	Block	3704				2	0.157
	Model	3704				2	0.157
Model Summary							
Step	−2 Log likelihood	Cox and Snell R Square			Nagelkerke R Square		
1	475,458 ^a^	0.010			0.014		
Variables in the Equation							
		B	S.E.	Wald	df	Sig.	Exp(B)
Step 1 ^b^	Coffee cups			3.624	2	0.163	
	Coffee cups (1)	−0.256	0.361	0.504	1	0.478	0.774
	Coffee cups (2)	−0.565	0.332	2.905	1	0.088	0.568
	Constant	0.838	0.299	7.850	1	0.005	2.312

^a^. Estimation terminated at iteration number 3 because parameter estimates changed by less than 001. ^b^. Variable(s) entered in step 1: Coffee cups.

## Data Availability

Data is contained within the article.
